# Supplementary material for: Activin A more prominently regulates muscle mass in primates than does GDF8
Source: Nat Commun. 2017 Apr 28;8:15153. doi: 10.1038/ncomms15153 (PMC5414365; doi:10.1038/ncomms15153)
Supplement: Supplementary Information — Supplementary figures and supplementary tables. [file ncomms15153-s1.pdf]

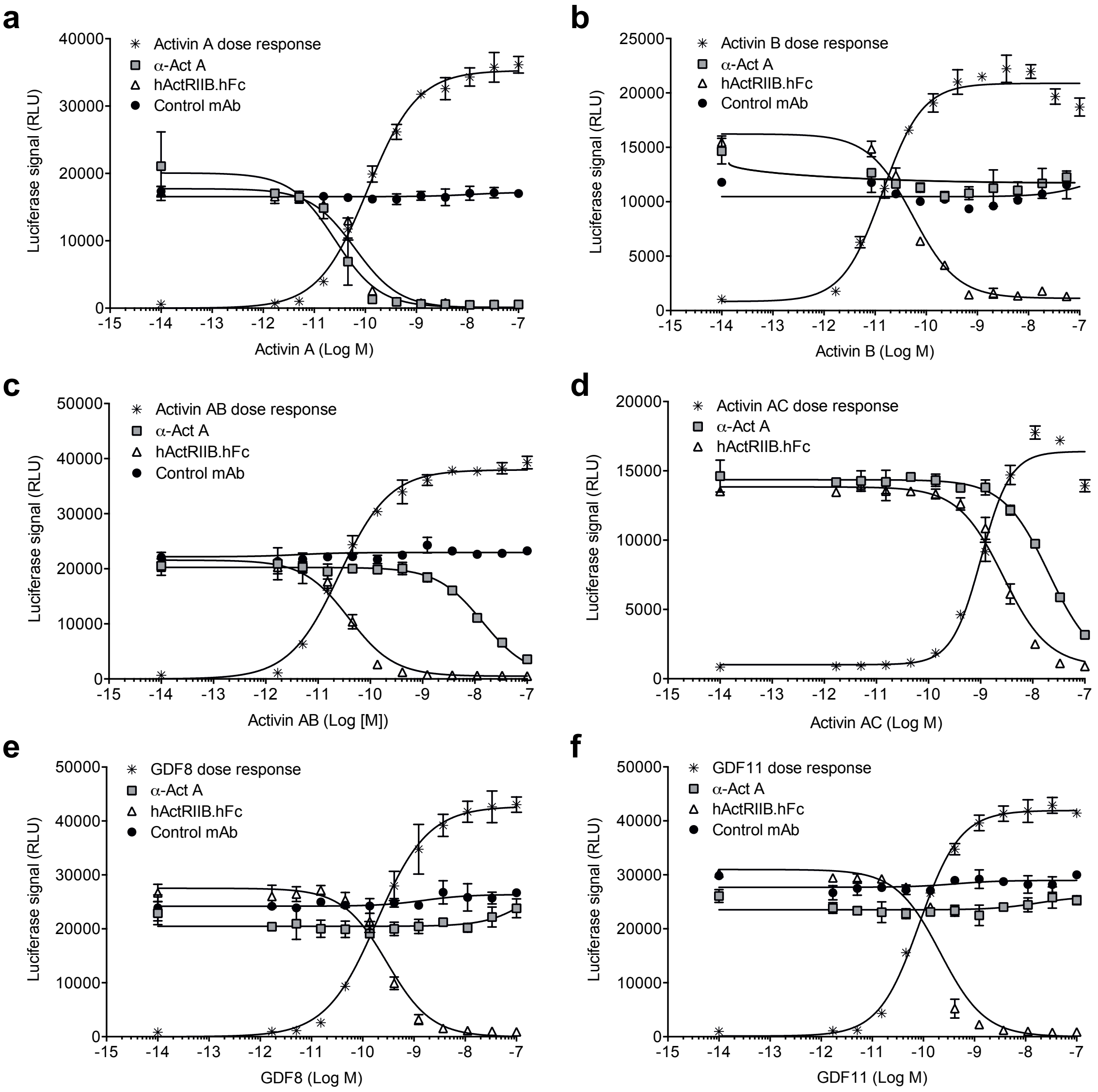


**Supplementary Figure 1**
**Biacore bioassay data for REGN2477 and ActRIIB.hFc**

Effects of α-activin A antibody (α-Act A; REGN2477) and ActRIIB.hFc on activin A, activin B, activin AB, activin AC, GDF8 and GDF11 stimulated Smad2/3 activity. Smad2/3 activity was recorded as relative luminescence units (RLU) from A204/Smad2/3/Luc reporter cells in the presence of increasing concentrations of activin A, activin B, activin AB, activin AC, GDF8 and GDF11. Smad2/3 activity was also recorded at a fixed concentration of (**a**) activin A, (**b**) activin B, (**c**) activin AB, (**d**) activin AC, (**e**) GDF8 or (**f**) GDF11 and increasing concentrations of control antibody (black circles), α-Act A (grey squares) and ActRIIB.hFc (white triangles). Data are shown as mean ± s.e.m. of three replicates.

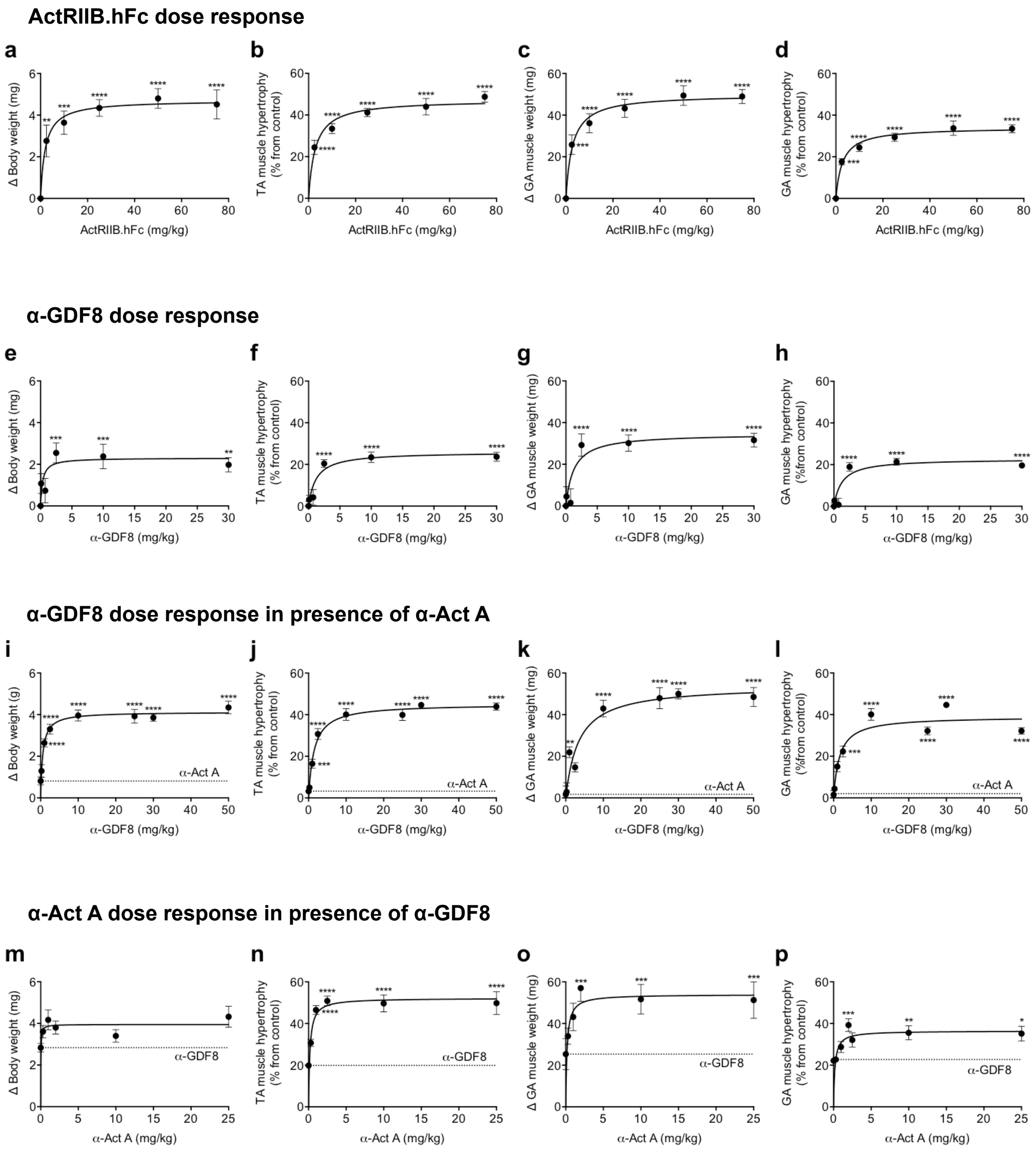


**Supplementary Figure 2**

**Effects of GDF8 and Activin A inhibition, compared to ActRIIB.hFc, on body and muscle weights**

Changes in body weight, TA muscle hypertrophy, GA muscle weight and GA muscle hypertrophy were recorded from 9-week-old, male SCID mice dosed for 21 days with increasing concentrations of ActRIIB.hFc (**a-d**), α-GDF8 (**e-h**), α-GDF8 in the presence of 10 mg/kg α-Act A (**i-l**), α-Act A in the presence of 10 mg/kg α-GDF8 (**m-p**). Body weight is expressed as the gram difference in final body weight over the control mean. Muscle mass or hypertrophy is expressed as gram difference or percentage change from the control mean, respectively. Data are shown as mean ± s.e.m. **a-h**: ***P*<0.01, ****P*<0.001, *****P*<0.0001 vs. control. **i-l**: ***P*<0.01, ****P*<0.001, *****P*<0.0001 vs. 10 mg/kg α-GDF8. **m-p**: **P*<0.05, ***P*<0.01, ****P*<0.001, *****P*<0.0001 vs. 10 mg/kg α-Act A. Statistical significance was calculated by one-way ANOVA with Bonferroni post-hoc test.

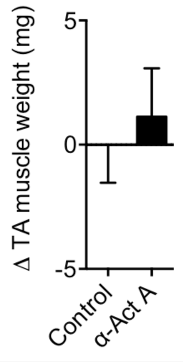


**Supplementary Figure 3**
**REGN2477 monotherapy does not significantly increase muscle mass in SCID mice**
Nine-week-old male SCID mice were treated for 21 days with 25 mg/kg of either REGN2477 or isotype control antibody (*n*=5 per group), after which TA muscle was excised and weighed. TA muscle weights are expressed as gram difference from the control mean. Data are shown as mean ± s.e.m.

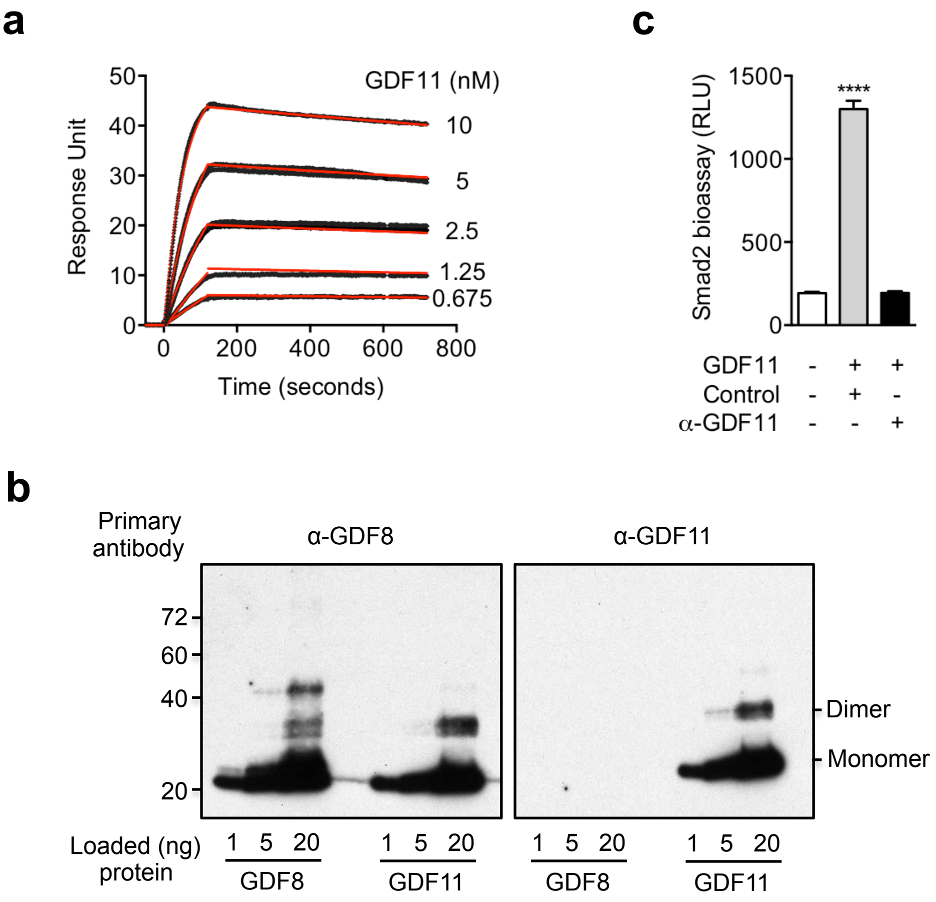


**Supplementary Figure 4**

**GDF11 antibody (α-GDF11) has high affinity and specificity to GDF11**

(**a**) Sensogram for GDF11 binding to α-GDF11 at 25 °C. Curves represent two-fold dilution series from 10 nM GDF11. (**b**) Western blot showing detection of GDF8 and GDF11 using a non-specific α-GDF8 mAb (left panel) and GDF11 using specific α-GDF11 mAb (right panel). (**c**) A204 SMAD2 bioassay showing 0.5 nM GDF11 pre-incubated with either 50 nM of α-GDF11 or control antibody for 30 min. Bioassay data in (**b**) are shown as mean ± s.e.m. of twelve replicates. ****$P$<0.0001 vs. control. Statistical significance was calculated by one-way ANOVA with Bonferroni post-hoc test.

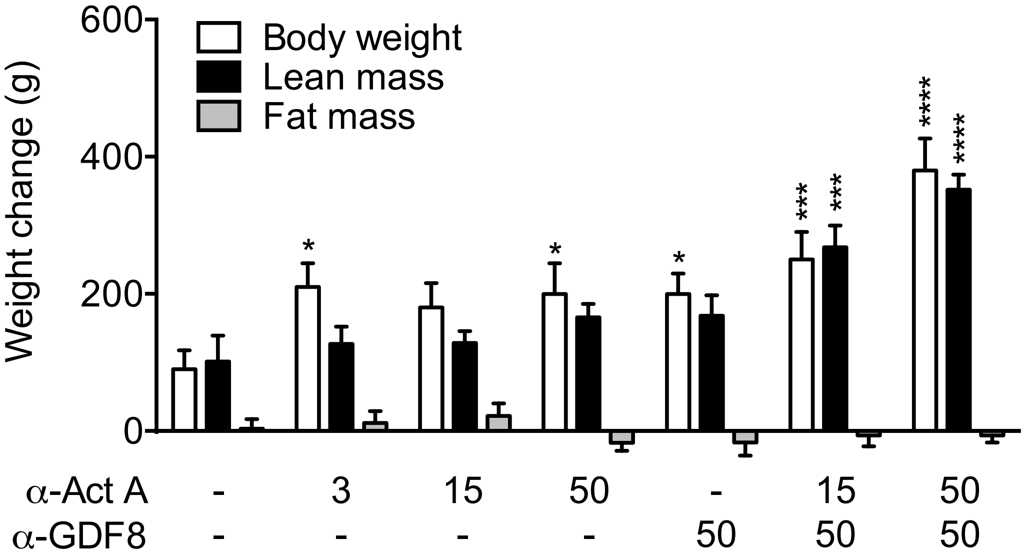


**Supplementary Figure 5**

**Body composition changes in monkeys treated with α-Act A and α-GDF8 for five weeks**

Change in body weight (white bars), lean body mass (black bars) and body fat mass (grey bars) in male and female cynomolgus monkeys (*n*=5 per gender/group). Indicated doses shown as mg/kg. Data are presented as the weight change in grams of the given parameter from the baseline and shown as mean ± s.e.m. *$P<0.05$, ***$P<0.001$, ****$P<0.0001$ vs. saline control. Statistical significance was calculated by one-way ANOVA with Bonferroni post-hoc test.

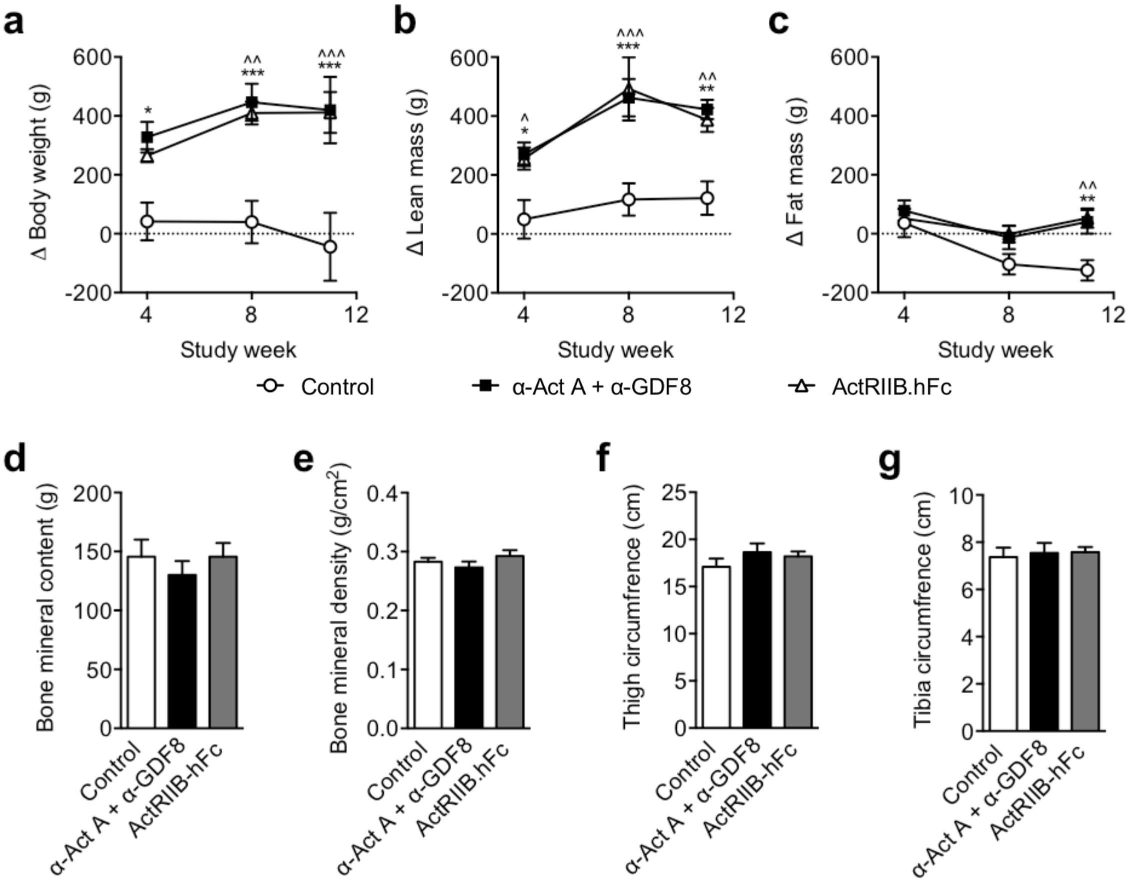


**Supplementary Figure 6**

**α-GDF8 and α-Act A treatment in monkeys for 11 weeks compared to ActrIIB.hFc**

Changes in body weight (**a**), lean mass (**b**) and fat mass (**c**) in male and female cynomolgus monkeys (*n*=3 per gender/group) dosed i.v. weekly for 11 weeks with 50 mg/kg of α-GDF8 and α-Act A or ActRIIB.hFc. One group of animals received saline as control. Bone mineral content (**d**), density (**e**), thigh circumference (**f**) and tibia circumference (**g**) were measured at week 8. Data are shown as mean ± s.e.m. *$P$<0.05, **$P$<0.01, ***$P$<0.001 for α-Act A and α-GDF8 vs. the respective weekly control, ^$P$<0.05, ^^$P$<0.01, ^^^$P$<0.001 for ActRIIB.hFc vs. respective weekly control. Statistical significance was calculated by two-way ANOVA with Bonferroni post-hoc test.

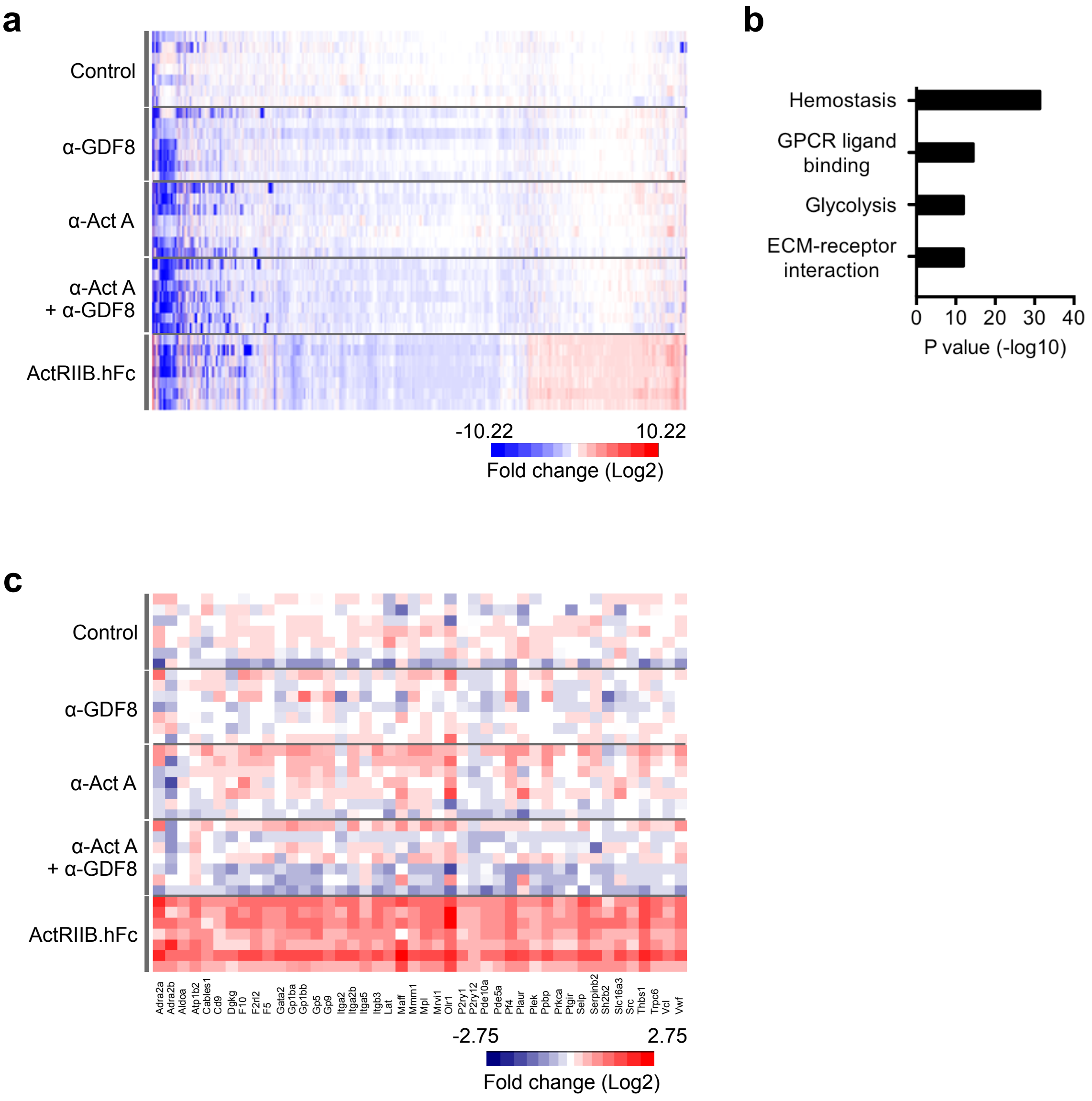


**Supplementary Figure 7**

**RNAseq of mouse spleen treated with α-GDF8 and α-activin A or ActRIIB.hFc**

(**a**) Heat map of the union of 2944 genes perturbed in spleen following a 10-day i.v. doing of α-GDF8, α-Act A, the combination of α-GDF8 and α-Act A or ActRIIB.hFc (*n*=7/group). (**b**) Illumina pathway analysis of the top regulated pathways from the ActRIIB.hFc group. (**c**) Heat map of significant regulated genes that are enriched in the hemostasis pathway listed in (**b**).

**Supplementary Table 1**

Kinetic binding parameters for $\alpha$-activin A and ActRIIB.hFc

| | | **Activin A** | **Activin B** | **Activin AB** | **Activin AC** | **GDF8** | **GDF11** |
|---|---|---|---|---|---|---|---|
| **$\alpha$-Act A** | **$K_D$ (M)** | 5.97E-12 | NB | 1.49E-09 | 2.07E-08 | NB | NB |
| | **T½ (min)** | 165 | NB | 1.3 | 0.3 | NB | NB |
| **ActRIIB.hFc** | **$K_D$ (M)** | <3.5E-12 | 6.40E-12 | 4.40E-12 | 5.90E-09 | 1.68E-11 | 1.40E-11 |
| | **T½ (min)** | >231 | 134 | 170 | 2.2 | 115 | 112 |

Association rate constants ($K_D$) and dissociative half-life ($T_{1/2}$) for $\alpha$-Act A and ActRIIB.hFc for the indicated ligands as measured by surface plasmon resonance at 25°C on a Biacore T200 instrument using a carboxymethyl dextran-coated CM4 sensor chip. NB = No binding at tested concentrations.

**Supplementary Table 2**

SMAD 2/3 bioassay data for $\alpha$-activin A and ActRIIB.hFc

| | **Activin A** | **Activin B** | **Activin AB** | **Activin AC** | **GDF8** | **GDF11** |
|---|---|---|---|---|---|---|
| **$\alpha$-Act A** | 2.60E-11 | NI | 1.40E-08 | 7.30E-09 | NI | NI |
| **ActRIIB.hFc** | 6.30E-10 | 5.50E-10 | 3.90E-10 | 8.50E-11 | 3.00E-10 | 2.10E-10 |

Determination of half-maximal inhibitory ($IC_{50}$) concentrations of $\alpha$-Act A and ActRIIB.hFc in a CAGAx12-luc SMAD2/3 signaling assay for the indicated ligands. All ligands show signaling activity in the tested cell line; NI = No inhibition for tested doses.

**Supplementary Table 3**

Effects of α-activin A and α-GDF8 or ActRIIB.hFc on glucose homeostasis in mice

| | Control | α-Act A + α-GDF8 | ActRIIB.hFc |
|---|---|---|---|
| **Blood glucose (mg/dL)** | 201 ± 6 | 220 ± 6 | 240 ± 6 *** |
| **Plasma insulin (ng/mL)** | 0.45 ± 0.09 | 0.68 ± 0.23 | 0.56 ± 0.09 |
| **Plasma glucagon (pg/mL)** | 23.8 ± 7.5 | 16.0 ± 4.4 | 17.5 ± 2.5 |
| **Plasma corticosterone (ng/mL)** | 88.2 ± 9.7 | 85.3 ± 5.5 | 76.9 ± 7.3 |
| **α-cell mass (mg)** | 0.13 ± 0.03 | 0.13 ± 0.01 | 0.12 ± 0.03 |
| **β-cell mass (mg)** | 1.5 ± 0.2 | 1.4 ± 0.3 | 1.6 ± 0.3 |

14-week-old male C57BL/6 mice were treated with a combination of α-GDF8 and α-Act A, ActRIIB.hFc or control antibody at 10 mg/kg each on Days 0 and 4 ($n$=6/group). Blood glucose and plasma hormones were measured at fed condition on Day 7. α-cell and β-cell masses were measured on Day 8 at fed condition. Data are shown as mean ± s.e.m. ***$P$<0.001 vs. control antibody by one-way ANOVA with Bonferroni post-hoc test.
